# Factors Associated With Insight Toward Illness in Patients With Bipolar Disorder Type 1 in Manic Episodes

**DOI:** 10.31083/AP44176

**Published:** 2025-06-24

**Authors:** Ya-Fan Chang, Si-Sheng Huang

**Affiliations:** ^1^Department of Psychiatry, Changhua Christian Hospital, 500 Changhua, Taiwan; ^2^Department of Post-Baccalaureate Medicine, College of Medicine, National Chung Hsing University, 402 Taichung, Taiwan

**Keywords:** adherence, bipolar disorder type 1, insight, manic episode, psychopathology

## Abstract

**Objective:**

Impaired insight presents a significant obstacle in the management of bipolar disorder. Research on the insight of patients with acute bipolar mania is lacking. The aim of this study was to provide understanding of patient insight in acute bipolar mania.

**Methods:**

A total of 52 inpatients who were diagnosed with bipolar disorder during a manic episode were included in the study. The Insight Scale for Affective Disorders (ISAD) was utilized, with high scores indicating poor insight. The Self-Appraisal of Illness Questionnaire (SAIQ) was used to assess patient attitudes and treatment experiences, with higher scores reflecting greater insight. Associated factors were identified through Pearson correlation and multiple linear regression analyses.

**Results:**

A low ISAD score was correlated with older age (*p* = 0.003), an extended duration of illness (*p* = 0.007), presence of a medical comorbidity (*p* = 0.012), and low scores on the Clinical Global Impressions-Severity (CGI-S) scale (*p* < 0.001), Clinical Global Impressions-Improvement (CGI-I) scale (*p* < 0.001), Young Mania Rating Scale (YMRS) (*p* < 0.001), and Frontal Assessment Battery (FAB) scale (*p *= 0.007). Multiple linear regression analysis revealed that the presence of a medical comorbidity (*p* = 0.031), low YMRS scores (*p* < 0.001), and low CGI-S scale scores (*p* = 0.044) were associated with low ISAD scores.

**Conclusions:**

Inpatients diagnosed with acute bipolar mania, a medical comorbidity, milder disease, and less severe manic symptoms had better insight. Patients with severe symptoms affecting motor activity, energy levels, sexual interest, sleep, and speech rates had less insight.

## Main Points

1. Individuals with acute bipolar mania, a medical comorbidity, mild disease, and less severe manic symptoms had greater insight.

2. Individuals with severe symptoms affecting motor activity, energy levels, sexual interest, sleep patterns, and speech rates had less insight during acute manic episodes.

3. Executive function may not be an independent predictor of disease insight but instead may play a role in mediating symptom severity and level of insight in patients recovering from acute mania.

## 1. Introduction

Bipolar disorder is a major psychiatric disorder that includes manic, depressive, or mixed-type episodes. This disorder causes disturbances in mood, sleep patterns, cognition, energy levels, behavior, and occupational and interpersonal function. In 2016, bipolar disorder was the 23rd leading cause disability worldwide [1]. Clinically, nonadherence to treatment is a major source of concern and affects patient outcomes. Among the factors contributing to treatment noncompliance, insufficient insight is the most important variable [2].

Insight into illness has been defined as a correct attitude toward a morbid change in oneself and an appropriate judgment, which could be accomplished by inference and change in oneself [3]. Full insight may include the patient’s attitude toward illness and illness awareness, and the patient could turn away from unusual experiences toward making a judgment about it and exploring its causes. Insight assessment is considered multidimensional and includes mental illness awareness, the need for treatment, and consequences associated with the illness [4].

Bipolar disorder type 1 and type 2 are characterized by differences in the duration and severity of their respective symptoms. Research indicates that individuals diagnosed with bipolar disorder type 1 often exhibit diminished cognitive functioning [5]. Furthermore, psychotropic medications, particularly antipsychotics, are more frequently used by patients with bipolar disorder type 1 [6]. These elements may influence both the awareness and prognosis of the condition. Because the high impact and challenge on clinical practice, individuals with bipolar disorder type 1 (BD) are the primary focus of this study, there are significant implications for clinical practice.

Previous studies have shown inconsistent results for relationship of demographics and clinical parameters to insight in BD. One study revealed that older age was associated with better insight [7], whereas another study did not reveal such an association [8]. Several studies revealed that female sex [9], younger age at onset [10], a longer duration of illness [9], higher numbers of hospitalization [10], and fewer manic episodes [11] were related to better insight. However, these studies focused primarily on patients with BD in the remission stage and lacked information on the acute manic stage. In comparative studies, patients with BD in the manic phase may tend to have poorer insight than those in the depressive or remission phases [12, 13]. Previous studies also revealed that insight improves as manic symptoms subside and indicated that insight is state dependent [11, 12, 14]. Poorer insight was associated with more severe manifestations of mania and psychotic symptoms, such as delusions and grandiosity [15].

Cognitive deficits, as evidenced by changes in current intelligence quotients, psychomotor and mental speed, attention, memory, visuospatial function, language/verbal fluency, executive function, and social cognition, are prevalent in individuals with BD [16]. Most studies revealed a significant positive correlation between insight and attention, as well as between insight and psychomotor skills, executive function, verbal fluency [10, 17], and memory [10]. However, in one study, researchers could not establish a significant association between insight and executive functions [18]. Executive function is likely the most extensively researched domain, and decreased executive function is most prevalent in patients with BD [19].

Numerous studies have indicated that patients’ insight tends to be significantly diminished during acute bipolar manic episodes than during periods of remission or depressive episodes [12, 13, 14]. However, there is a paucity of research specifically exploring the clinical insight of individuals experiencing acute bipolar mania. Consequently, it is imperative to investigate the factors that may influence awareness and insight within this patient population. The relationships among various patient characteristics, such as clinical demographics, illness severity, symptomatology, cognitive functioning, and insight into bipolar disorder during manic episodes, have not been consistently documented. Additionally, cultural context may play a crucial role in shaping both public and patient perceptions and attitudes toward the disorder. The help-seeking behaviors of patients are likely affected by conceptualizations and interpretations of mental health conditions that vary across different regions and cultures [20]. The aim of this study is to explore the disease insight of individuals in Taiwan, which is situated in Asia. The primary objective of this study was to identify the associations of sex, age, age at onset, duration of illness, frequency of hospitalization, clinical severity, symptom severity, and cognitive function with clinical insight among patients with bipolar disorder type 1 during manic episodes. Furthermore, the aim of this study is to identify variables that may mediate the relationships between these factors and insight. We hypothesize that patients’ insight will be significantly correlated with the severity of manic episodes, their associated psychopathology, and patients’ cognitive functions, particularly executive functioning.

## 2. Materials and Methods

### 2.1 Inclusion and Exclusion Criteria

We recruited 52 patients who experienced an acute manic episode of BD in the psychiatric ward of a medical center in Taiwan between December 2017 and November 2019. The inclusion criteria were aged 18–75 years, experienced an acute manic episode of BD, hospitalized for at least 2 weeks and able to be interviewed. Patients were diagnosed using the fifth edition of the Diagnostic and Statistical Manual of Mental Disorders (DSM-5) [21] and on the basis of a chart review by a senior psychiatrist. In order to ensure the patient’s cooperation during the interview, we choose to admit the case after the patient’s condition is relatively stable after appropriate treatment. Assessments were conducted after an average hospitalization period of 21.71 days, with a standard deviation of 10.43 days, and a range of 14 to 56 days. Individuals with intellectual disabilities, mild or major neurocognitive disorders, substance use disorders, or other organic mental disorders were excluded from the study. During the study period, all participants received daily treatment as usual. This study was cross-sectional in nature. The Institutional Review Board of Changhua Christian Hospital approved this study (Approval No: 171108, Date: December 1, 2017). Informed consent was obtained from all the patients who agreed to participate in the study or their legal guardians.

### 2.2 Insight Assessment

Given that this study encompasses inpatients diagnosed with acute mania, it is imperative not only to evaluate the patient’s clinical insight but also to thoroughly assess their awareness of their symptoms. Consequently, we selected the Insight Scale for Affective Disorders (ISAD) [22] for objective evaluations. The ISAD is widely used in clinical research to measure insight levels in patients with mood disorders [12, 13, 14]. The ISAD contains 2 sections (general and awareness) with a total of 17 items, each of which is rated on a six-point scale: 0 (absence of symptoms), 1 (full awareness), and 5 (absence of awareness), with high scores indicating low awareness and insight. The general section (items 1–3) evaluate the patient’s awareness of their BD, perceptions about the efficacy of medications, and perceived social consequences of BD. The awareness section (items 4–17) evaluates the patient’s awareness of individual manic symptoms of BD. Internal consistency was satisfactory across all items, as indicated by a Cronbach’s alpha coefficient of 0.88. Additionally, moderate to strong correlations were observed among the scores on the ISAD, various measures of insight, and results from certain clinical assessments, thereby reinforcing the validity of the tool [22]. In this study, the Cronbach’s alpha value was 0.844. Items 4–17 of the ISAD evaluate a patient’s awareness of their symptoms. The relationship between individual manic symptoms and level of insight has been rarely investigated in other studies.

### 2.3 Clinical Assessment

We planned to assess the subjective insight and attitude from the patient’s perspective, so a self-administered scale, the Self-Appraisal of Illness Questionnaire (SAIQ) [23], was chosen. It has 17 items and is designed to capture the patients’ attitudes toward their mental illness and treatment experiences. The results of the questionnaire may also represent the patient’s clinical insight. The SAIQ contains three-dimensional factors, such as worry, need for treatment, and presence/outcome of illness, to investigate the patient’s awareness of the psychosocial impact of having a mental illness, attitudes toward treatment, and level of awareness of having a mental disorder, respectively. Participants provide responses on a 4-point scale, with ratings ranging from 0 indicating strongly disagree to 3 indicating strongly agree, and higher SAIQ scores indicate greater awareness of and insight into the illness. Internal consistency was satisfactory across all items, as indicated by a Cronbach’s alpha coefficient of 0.867. Furthermore, the three subscales, along with the total score, were significantly correlated with an alternative research-rated insight scale. In this investigation, the Cronbach’s alpha value was 0.752.

Symptoms associated with manic episodes in BD patients were assessed using the Young Mania Rating Scale (YMRS) [24]. The YMRS is an 11-item scale administered by clinicians in which the cumulative score ranges from 0 to 60. The specific items evaluated by the YMRS include (1) elevated mood, (2) increased motor activity and energy, (3) sexual interest, (4) sleep patterns, (5) irritability, (6) speech rate and volume, (7) language and thought processes, (8) content of plans, (9) disruptive or aggressive behavior, (10) appearance, and (11) insight. A total score ≤6, 7–20, or >20 indicates euthymia, a milder manic state, or an acute manic state, respectively. We used the Clinical Global Impressions-Severity (CGI-S) and Clinical Global Impressions Scale-Improvement (CGI-I) scales [25] to evaluate overall illness severity and improvement. A higher CGI-S score indicates greater illness severity, and a lower CGI-I score represents greater improvement after treatment. General cognitive and executive functions were measured using the Montreal Cognitive Assessment (MoCA) [26] and frontal assessment battery (FAB), respectively [27].

### 2.4 Statistical Analysis

We presented demographic data as frequencies, percentages, and means ± standard deviations. Data were analyzed using SPSS version 22.0 (IBM SPSS Corp.; Armonk, NY, USA). The correlation between the ISAD score and continuous variables, such as age and duration of illness, was assessed through the Pearson’s correlation coefficient. Additionally, independent *t* tests were employed to evaluate differences in ISAD scores across categorical groups, such as gender and marital status. As some parameters failed to meet the assumptions (normal distribution) necessary for conducting Pearson’s correlation, we used Spearman’s correlation to test the correlation between ISAD and years of education, age at onset, number of hospitalization, CGI-S, CGI-I, YMRS, and MoCA. Multiple linear regression analyses were used to identify variables associated with clinical insight. The G*Power software (version 3.1.9.7; Heinrich Heine University Dusseldorf, Dusseldorf, Germany) [28] was used to estimate the requisite total sample size for our study. We established a type I error rate (α) of 0.05, a statistical power of 0.8, a predictor count of 9, and a squared multiple correlation coefficient (R^2^) of 0.16. The calculation indicated that a sample size of 91 participants would be necessary; however, we were only able to recruit 52 patients. Consequently, the analysis revealed that the statistical power of the test was approximately 0.47. To improve the statistical power of the test, we conducted a correlation analysis to identify variables associated with the dependent variables, subsequently removing highly correlated independent variables. Ultimately, we selected age, medical comorbidities, CGI-S score, YMRS score, and FAB score for further analysis. These five variables were then incorporated into a multiple linear regression analysis using the enter method. To define the possible mediation effect, we used the following criteria: (1) the independent variable must be significantly associated with the possible mediator in the first linear regression; (2) the independent variable must be significantly associated with the dependent variable in the second equation; and (3) the mediator must be significantly associated with the dependent variable in the third linear regression. Perfect mediation is noted if the independent variable does not affect the dependent variable when the mediator is controlled. If the independent variable is significantly associated with the dependent variable with a smaller coefficient, this suggests a partial mediation and may have multiple mediating factors [29]. For all the statistical tests, a *p* value < 0.05 indicated statistical significance.

## 3. Results

### 3.1 Demographic and Clinical Characteristics

Table 1 shows the demographic and clinical characteristics of the participants. Fifty percent of the participants were male, and 50% of the participants were female. The mean age of the participants was 48.25 ± 12.74 years. The average duration of illness was 20.1 ± 12.11 years. The average number of hospitalizations was 8.15 ± 4.48. The mean total scores on the YMRS and CGI-S scale were 12.90 ± 9.77 and 3.94 ± 0.83, respectively. The mean total MoCA score was 21.17 ± 4.74.

**Table 1.** **Demographics and clinical characteristics of the patients**.

		Patients (n = 52)
Sex, male (%)		26 (50)
Age, years		48.25 ± 12.74
Family history, yes (%)		34 (65.4)
Education, years		11.88 ± 2.80
Marital status, no (%)		17 (32.7)
Employment, yes (%)		27 (51.9)
Age at onset, years		28.15 ± 10.24
Length of illness, years		20.1 ± 12.11
Number of hospitalizations		8.15 ± 4.48
Medical comorbidity, yes (%)		28 (53.8)
	Hypertension	15 (28.8)
	Diabetes mellitus	11 (21.2)
	Hyperlipidemia	8 (15.4)
CGI-S		3.94 ± 0.83
CGI-I		2.52 ± 0.61
YMRS		12.90 ± 9.77
MoCA		21.17 ± 4.74
FAB		12.21 ± 3.15
SAIQ total		28.44 ± 9.56
SAIQ- Worry		8.92 ± 5.95
SAIQ- Need for treatment		10.40 ± 2.55
SAIQ- Presence of illness		9.12 ± 2.75
ISAD		30.29 ± 12.12

Notes: Data are presented as frequencies, percentages, and mean ± standard deviations. CGI-S, Clinical Global Impressions Scale-Severity; CGI-I, Clinical Global Impressions Scale-Improvement; YMRS, Young Mania Rating Scale; MoCA, Montreal Cognitive Assessment; FAB, Frontal Assessment Battery; SAIQ, Self-Appraisal of Illness Questionnaire; ISAD, Insight Scale for Affective Disorders. SAIQ comprises worry, need for treatment, and presence of illness factors.

### 3.2 Correlation Analysis of Clinical Parameters and Insight (ISAD)

Table 2 lists the results of the Pearson’s and Spearman’s correlation analyses between the patients’ clinical characteristics and ISAD scores (clinical insight). Lower ISAD scores were significantly correlated with older age, the presence of a medical comorbidity, and a longer duration of illness. In terms of illness severity, lower CGI-S scores, lower CGI-I scores, and lower YMRS scores were significantly correlated with lower ISAD scores. With respect to cognitive function, higher FAB scores were significantly correlated with higher ISAD scores. With respect to the self-rated questionnaire, higher scores on the need for treatment subscale of the SAIQ and the presence of illness subscale of the SAIQ were correlated with lower ISAD scores.

**Table 2.** **Correlation between clinical characteristics of patients and total score of ISAD**.

Demographics of the patients		ISAD (mean ± SD)	r^a,b^ or T^c^ or F^d^	*p*-value
Age, years			−0.407^a^	0.003**
Gender				
	Male	31.69 ± 11.03	0.833^c^	0.409
	Female	28.88 ± 13.19		
Education, years			0.065^b^	0.646
Family history				
	Yes	30.44 ± 12.98	−0.124^c^	0.902
	No	30.00 ± 10.65		
Marital status				
	Single	35.71 ± 13.12	2.821^d^	0.069
	Married	27.18 ± 11.44		
	Divorced	29.57 ± 8.58		
Employment				
	Yes	28.81 ± 13.44	0.910^c^	0.367
	No	31.88 ± 10.55		
Medical comorbidity				
	Yes	26.46 ± 10.01	2.592^c^	0.012*
	No	34.75 ± 13.02		
Age at onset, years			−0.027^b^	0.850
Duration of illness (years)			−0.369^a^	0.007**
Number of hospitalizations			−0.305^b^	0.058
CGI-S			0.731^b^	<0.001***
CGI-I			0.507^b^	<0.001***
YMRS			0.809^b^	<0.001***
MoCA			0.190^b^	0.178
FAB			0.367^a^	0.007**
SAIQ total			−0.363^a^	0.008**
SAIQ- Worry			−0.235^a^	0.094
SAIQ- Need for treatment			−0.403^a^	0.003**
SAIQ- Presence of illness			−0.381^a^	0.005**

Notes: *p < 0.05; **p < 0.01; ***p < 0.001; ^a^Pearson’s correlation analysis; ^b^Spearman’s correlation analysis; ^c^independent *t* test; ^d^analysis of variance. SAIQ comprises worry, need for treatment, and presence of illness factors.

### 3.3 Significant Associations between Medical Comorbidity History, CGI-S Score, YMRS Score and ISAD Score

Multiple linear regression analyses revealed that the presence of a medical comorbidity and CGI-S and YMRS scores were significantly associated with the total ISAD score, and the model explained 71.0% of the total variance (F = 25.936, *p* < 0.001), as shown in Table 3.

**Table 3.** **Multiple linear regression for insight (ISAD)**.

Variable	β	*p-*value
Age	−0.045	0.640
Comorbidity (0 = no, 1 = yes)	−0.203	0.031*
CGI-S	0.266	0.044*
YMRS	0.553	<0.001***
FAB	0.005	0.954
Dependent variable	Total score of ISAD	
Total adjusted variance explained	71.0%, F = 25.936***, *p* < 0.001	

Notes: Multiple linear regression analyses; *p < 0.05; ***p < 0.001.

### 3.4 Role of Executive Function in Mania Severity and Insight

**Fig. 1.**
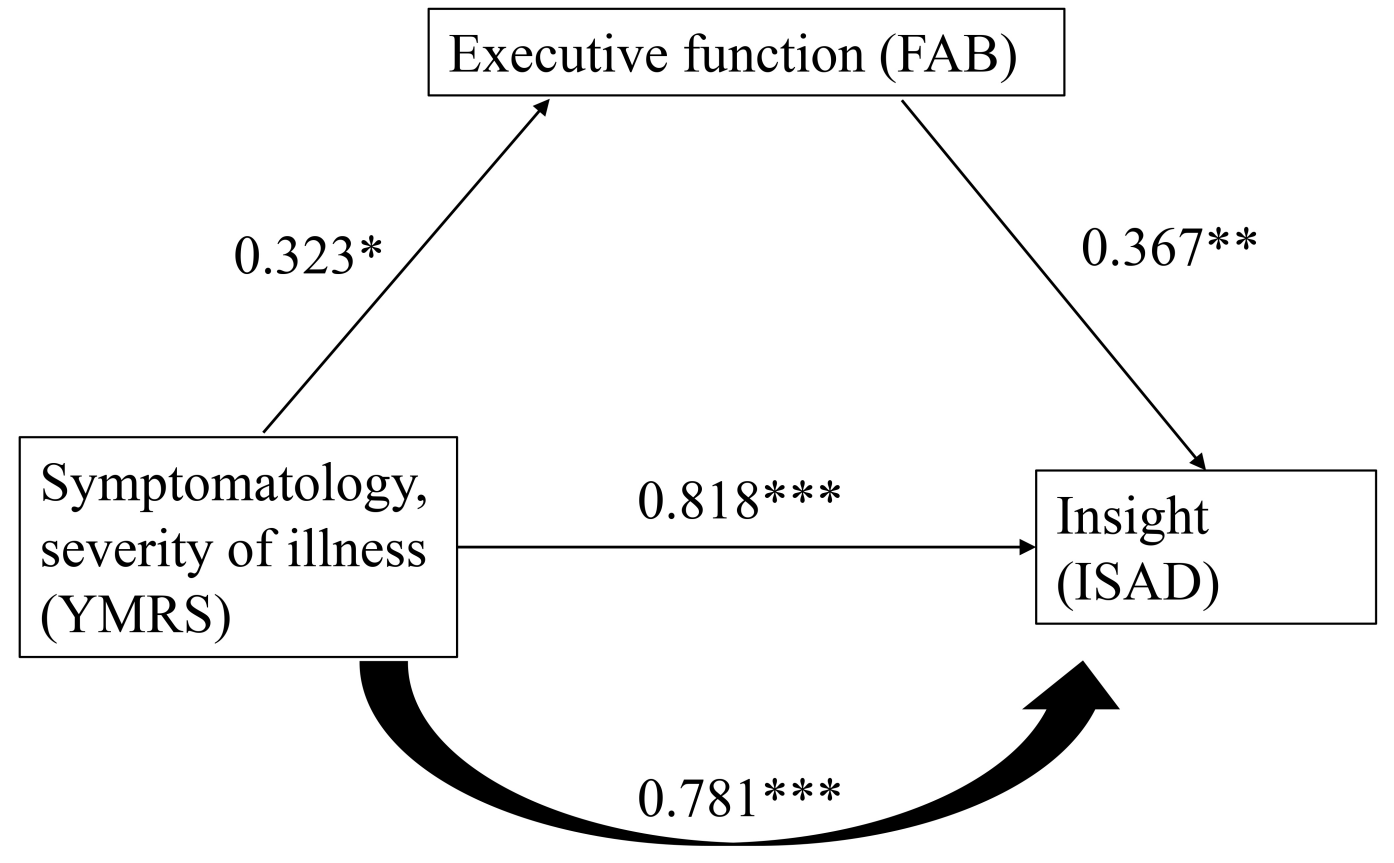
**The relationship of symptomatology, executive function, and insight**. The figure was based on results of linear regression and the coefficients between variables. *p < 0.05; **p < 0.01; ***p < 0.001. A higher ISAD score indicates poorer insight.

As shown in Fig. 1, YMRS (mania severity) scores were significantly associated with FAB (executive function) and ISAD (insight) scores. When both YMRS and FAB scores were used as independent variables and ISAD scores were used as dependent variables in the regression analysis, the coefficient for the YMRS (β = 0.781, *p* < 0.001) was significant but smaller than that for the YMRS alone (β = 0.818, *p* < 0.001) in the linear regression. Executive function may have a mediating effect on the relationship between manic episode severity and insight.

### 3.5 Greater Severity of Certain Symptoms was Correlated With Lower Overall Insight

We conducted a partial correlation analysis of individual items from the YMRS, SAIQ, and the ISAD after controlling for scores on the FAB and MoCA, as presented in Table 4. Specifically, items 2 (increased motor activity and energy), 3 (sexual interest), 4 (decreased need for sleep), 6 (speech rate and amount), and 11 (insight) exhibited significant negative correlations with the total SAIQ score. In terms of objective insight, as measured by the ISAD, all 11 symptoms listed in the YMRS were significantly correlated with the total ISAD score, indicating that greater symptom severity was associated with less insight.

**Table 4.** **Correlation between individual symptoms and insight (SAIQ and ISAD)**.

YMRS item	Total	SAIQ			Total	ISAD	
		Worry subscale	Need for treatment	Presence of illness		General subscale	Awareness subscale
1. Elevated mood	−0.261	−0.228	−0.246	−0.181	0.707***	0.354*	0.709***
2. Increased motor activity and energy	−0.280*	−0.165	−0.329*	−0.305*	0.720***	0.310*	0.736***
3. Sexual interest	−0.321*	−0.333*	−0.187	−0.218	0.487***	0.183	0.505***
4. Sleep	−0.296*	−0.171	−0.452**	−0.234	0.406**	0.244	0.397**
5. Irritability	0.027	−0.040	0.020	0.160	0.325*	−0.039	0.380**
6. Speech rate and amount	−0.295*	−0.218	−0.294*	−0.274	0.698***	0.320*	0.708***
7. Language–thought	−0.185	−0.129	−0.218	−0.158	0.649***	0.230	0.676***
8. Content of the plans	−0.203	−0.124	−0.239	−0.213	0.630***	0.270	0.644***
9. Disruptive-aggressive behavior	−0.127	−0.073	−0.160	−0.131	0.317*	0.104	0.332*
10. Appearance	−0.144	−0.166	−0.054	−0.088	0.294*	0.014	0.330*
11. Insight	−0.589***	−0.411**	−0.635***	−0.558***	0.569***	0.692**	0.462**
Total score	−0.345*	−0.255	−0.374**	−0.294*	0.797***	0.370**	0.808***

Notes: *p < 0.05; **p < 0.01; ***p < 0.001; Partial correlation, executive function (FAB), and cognitive function (MoCA) were controlled. SAIQ comprises worry, need for treatment, and presence of illness factors. ISAD comprises general and awareness subscales.

## 4. Discussion

### 4.1 Correlation of Insight With Demographic and Clinical Variables

Some studies revealed a positive correlation between insight and age [7, 17], whereas others revealed no correlation [12]. Older age and a longer duration of illness were correlated with better insight in this small-sample study. Greater insight was also correlated with frequent hospitalizations (*p *= 0.079). We speculate that older patients and patients with a longer duration of illness may be more frequently admitted to the hospital and more likely to have better knowledge of their disease. Patients may become more knowledgeable based on their experiences in dealing with the disease and therefore more aware of differences between normal life and manic episodes. The findings of this study must be interpreted carefully owing to the small sample size and cross-sectional nature of the study.

The Pearson correlation analysis revealed a significant association between the presence of medical comorbidities and enhanced disease insight among patients. Given that we specifically excluded individuals with alcohol and substance use disorders, we deduce that patients with comorbid physical conditions, excluding alcohol and substance use disorders, may exhibit greater insight. Furthermore, it is plausible that adherence to medication regimens for cooccurring physical illnesses may positively influence both medication compliance and insight into mental health conditions.

Severe manic symptoms are significantly correlated with poor insight [10]. Previous studies revealed that insight into BD is likely state-dependent and that, compared with mixed type, depressive, and remission episodes, acute manic episodes were significantly associated with poorer insight [11, 12, 13]. Classification of the patient’s current mood episode status into acute manic, subacute manic, remitted, or depressive status is more appropriate when investigating the insight of patients with BD. The clinical insight into BD could be examined based on the patient group’s specific mood status. In the current study, patients’ insight worsened with symptom severity in the acute manic phase. However, a patient’s insight did not worsen with worsening executive function, as expected. In contrast, as executive function improves, the patient’s insight into illness decreases, indicating that poorer executive functioning does not predict poorer illness awareness in either the acute or subacute state of bipolar mania. This finding may represent a unique phenomenon in patients who have recently partially recovered from acute mania but are not in complete remission. Varga *et al*. [18] reported that this phenomenon reflects the absence of a relationship between neuropsychological abnormalities and a lack of insight into BD, or it may represent an indirect manifestation of frontal lobe dysfunction. Certain cognitive function deficits lead to worsening of the patient’s insight, and other executive functions compensate for impaired functions. However, this explanation has not been properly studied and confirmed. Some researchers [30, 31] reported that cognitive deficits were not correlated with impaired insight.

While executive function may not serve as an independent variable influencing insight in patients with bipolar disorder (BD) during the acute phase, it appears to mediate the relationship between the severity of mania and insight (see Fig. 1). The data illustrated in Fig. 1 indicates a correlation between elevated levels of manic symptomatology and illness severity with increased ISAD scores. Futhermore, a positive correlation was observed between enhanced executive function and higher ISAD scores, which in turn suggested a poorer insight. The findings suggest that as executive function improves, clinical insight deteriorates. These observations are particularly relevant for patients experiencing acute bipolar mania during their recovery phase, wherein symptoms are not entirely remitted. During this period, patients may exhibit inflated self-esteem, overconfidence in managing various situations and a lack of concern regarding daily challenges. Symptoms such as mildly elevated energy levels, an exaggerated sense of well-being, and heightened self-confidence remain evident. Simultaneously, cognition transitions from disorganized and incoherent, as evidence by tangential speech patterns, to a state characterized by mild distractibility, circumstantiality, and improved communicative abilities as the patient recovers from the acute manic episode. Consequently, the presence of more severe manic symptoms, coupled with relatively enhanced executive function, may contribute to the observed decline in patient insight.

### 4.2 Associated Factors of Insight

In the context of regression analysis, the presence of medical comorbidities, the overall severity of a patient’s illness, and the severity of psychopathology are well known for their association with level of insight. Pearson’s correlation analysis revealed that these three factors were significantly correlated with insight, and regression analyses revealed that these three factors were independent predictors of insight. To our knowledge, this study represents the first investigation to establish a link between medical comorbidities and greater insight. However, importantly, the sample size in this study is very small, thus further research is needed to validate these findings. Additionally, individuals with more severe disease and psychopathology tend to demonstrate lower levels of insight, a result that aligns with previous studies [12, 13, 18]. Furthermore, other clinical characteristics, such as age and executive function, were not significantly associated with clinical insight.

### 4.3 Correlation of Symptomatology and Insight

Insight and psychopathology is negatively correlated in patients with acute manic episodes. Psychopathology can be regarded as directly related to insight [15]. The advantage of this study is the use of self-assessment tools such as the SAIQ and objective assessment tools such as the ISAD. The SAIQ not only reflects attitudes but also represents subjective insight into mental illness [23]. We believe that the need for treatment subscale (attitudes toward psychiatric treatment) and the presence/outcome of illness subscale (awareness of having a disorder) are the most appropriate for assessing patients’ insight into their illness [23]. We chose the SAIQ for analysis because the ISAD and the YMRS share overlapping items.

Patients with severe symptoms that affected their motor activity, energy levels, sexual interest, sleep, and speech rate and amount had lower overall insight according to the SAIQ total score (Table 4). In addition, patients with greater insight into psychiatric treatment (need for treatment subscale) had less severe symptoms that affected their motor activity, energy levels, sleep, and speech rate and amount. Patients with better awareness of their BD (presence/outcome of illness subscale) had less severe symptoms that affected their motor activity and energy levels. Thus, the lower the severity of the manic symptoms (total YMRS score), the greater the patient’s insight into BD and likelihood of accepting treatment. No significant correlation was found between overall symptom severity (total YMRS score) and patients’ awareness of the social consequences of mood disorders (worry domain). Our findings were partially comparable with those of Silva *et al*. [32], who reported that poor global insight among patients with BD is correlated with more severe changes in mood, speech, thought structure, and agitation/energy, particularly increased psychomotor activity. Increased activity and energy levels, which are characteristic features of BD, were strongly correlated with the severity of manic symptoms [33]. Moreover, increased motor activity and energy levels were strongly correlated with various dimensions of attitude and levels of insight, which was consistent with the abovementioned research indicating the importance of such symptoms of BD.

The present study has several limitations. First, the sample size was relatively small, which may have resulted in insufficient statistical power and an increased likelihood of “false negative” outcomes, thereby hindering the detection of originally significant differences. Consequently, the null findings regarding the influence of various variables on insight may stem from this lack of power. Second, the cross-sectional design employed in this research restricts the ability to investigate the dynamic nature of insight and the causal relationships among variables, as it does not allow for the observation of changes over time. Third, the generalizability of the findings is limited, as participants were exclusively recruited from an acute psychiatric ward. Insight into illness among patients receiving different treatment modalities, such as outpatient services, chronic inpatient care, and psychiatric community support, may vary significantly. Thus, this study primarily elucidates the factors associated with clinical insight in inpatients diagnosed with bipolar disorder type 1. Nevertheless, it offers valuable reference material for future research endeavors, particularly given that the literature predominantly addresses insight during remission periods, with limited focus on individuals experiencing acute mania. Fourth, there may be recruitment bias, as patients exhibiting poorer insight might have been less inclined to participate in the study. Fifth, the inability to gather supplementary information from family members is also a limitation, as certain data, including the duration of illness and frequency of episodes, may be underestimated due to memory lapses. Sixth, patients with and without psychotic features were not separated for the statistical analysis despite the potential correlation between insight and psychotic symptom severity [15]. Finally, we did not control for confounding factors related to medication effects, such as those induced by mood stabilizers and antipsychotics, which could significantly influence cognitive functions and insight [34, 35]. Future research specifically designed to assess the confounding effects of pharmacological treatments is warranted to address this intricate issue.

## 5. Conclusions

In this study of acute bipolar manic patients in the recovery process, the presence of a medical comorbidity, mild disease and less severe manic symptoms were independently associated with greater insight. Executive function may not be independently associated with disease insight but may instead have a mediating effect on manic symptom severity and insight. Patients with severe symptoms that affect motor activity, energy levels, sexual interest, sleep, and speech rate and amount have overall poor insight. These results highlight the importance of adequate treatment of manic symptoms as a first step toward managing poor insight in patients with BD during manic episodes. Classifying mood episodes into acute manic, subacute manic, remitted, or depressive states may be more appropriate when assessing the insight of patients with BD.

## Data Availability

The data that support the findings of this study are available from the corresponding author upon reasonable request.
